# Chinese herbal extract reduces Amyloid-β induced neurotoxicity through inhibiting NF-κB signaling pathway in neuronal cells

**DOI:** 10.1186/1750-1326-7-S1-S28

**Published:** 2012-02-07

**Authors:** Xuehua Wu, Yan Yu, Liang Zhou, Hecheng Wang, Huan Yang, Kaiyin Zhong, Ximeng Zhang, Hui Zhang, Dongsheng Fan, Dehua Chui

**Affiliations:** 1 Peking University Neuroscience Research Institute, Dept. of Neurology, Peking University Third Hospital, Beijing 100191, China

## 

Amyloid-β (Aβ) peptide, which can invoke a cascade of inflammatory responses, is considered to play a causal role in the development and progress of Alzheimer’s disease (AD). Xylocoside G (XG) is an active compound isolated from a traditional Chinese medicinal plant, Itoa orientalis. We have previously reported that XG has neuroprotective effects, with the mechanism yet unknown. In this study, we investigated the possible mechanisms underlying neuroprotection of XG against Aβ-induced toxicity in SH-SY5Y cells and primary neurons. Pretreatment with XG significantly attenuated the cell viability reduction induced by Aβ exposure in a dose dependent manner which was testified by 3-[4, 5-Dimethylthiazol-2-yl]-2, 5-diphenyltetrazolium bromide (MTT) and Lactate dehydrogenase (LDH) release assay. In addition, pretreatment with XG reversed the effect of Aβ on Bax and Bcl-2 expression and repressed Aβ-induced caspase-3 activation, suggesting that the neuroprotective effect of XG is associated with apoptosis regulation. Neuroinflammation has been implicated in Aβ-induced neuronal death. XG significantly attenuated Aβ-stimulated release of inflammatory factors such as tumor necrosis factor-α (TNF-α), interleukin-1β (IL-1β) and prostaglandin E_2_ (PGE_2_). It also downregulated the expression of cyclooxygenase-2 (COX-2) in SH-SY5Y cells. Further molecular mechanism studies demonstrated that XG inhibited Aβ-induced NF-κB p65 translocation, which was probably the result of inhibition of JNK phosphorylation but not ERK or p38 MAPK pathway by XG. This is the first study to demonstrate that XG protects SH-SY5Y cells against Aβ-induced inflammation and apoptosis through down-regulating NF-κB signaling pathways.

